# Angiographic Complete versus Clinical Selective Incomplete Percutaneous Revascularization in Heart Failure Patients with Multivessel Coronary Disease

**DOI:** 10.1155/2020/9506124

**Published:** 2020-07-27

**Authors:** Chieh-Yu Chang, Chun-Chi Chen, I-Chang Hsieh, Ming-Jer Hsieh, Cheng-Hung Lee, Dong-Yi Chen, Ming-Lung Tsai, Ming-Yun Ho, Jih-Kai Yeh, Yu-Chang Huang, Yu-Ying Lu, Chao-Yung Wang, Shang-Hung Chang, Ming-Shien Wen

**Affiliations:** Division of Cardiology, Department of Internal Medicine, Chang Gung Memorial Hospital, Chang Gung University College of Medicine, Address: No. 5, Fu-Hsing Street, Kwei-Shan, Taoyuan, Taiwan

## Abstract

**Background:**

Patients with multivessel disease (MVD) often pursue complete revascularization (CR) during percutaneous coronary intervention (PCI) to improve prognosis. However, angiographic CR is not always feasible and is associated with some procedure-related complications in heart failure (HF) patients with MVD. Clinical selective incomplete revascularization (IR) may be reasonable for these high-risk patients, but its role in long-term outcomes remains uncertain.

**Methods:**

Six hundred patients with HF and MVD submitted to PCI were enrolled. Major adverse cardiac events (MACEs) were defined as a composite of recurrent myocardial infarction, any revascularization, and all-cause mortality at 5 years.

**Results:**

During a mean follow-up period of 3.7 ± 1.9 years, there was no significant difference in 5-year MACEs between selective IR and successful angiographic CR in HF patients with MVD. However, patients who failed CR had a significantly greater incidence of 5-year MACEs than those in the other two groups (failed CR: 46.4% vs. selective IR: 27.7% vs. successful CR: 27.8%, *p* < 0.001).

**Conclusions:**

Long-term outcomes of selective IR were comparable with those of successful angiographic CR in HF patients with MVD. However, patients that failed CR showed 2.53-fold increased risk of MACEs compared to patients undergoing either selective IR or successful angiographic CR. A more comprehensive planning strategy should be devised before PCI in HF patients with MVD.

## 1. Introduction

Heart failure (HF) owing to ischemic cardiomyopathy is currently an epidemic and an increasing health care burden due to high mortality and poor prognosis [1, 2]. Guideline-directed medical therapy (GDMT), which includes the use of angiotensin-converting enzyme inhibitors (ACEi) or angiotensin receptor blockers (ARB), beta-blockers, and mineralocorticoid receptor antagonists (MRA), has greatly improved the survival rate of patients with reduced ejection fraction (EF) [2]. Moreover, compared with GDMT alone, GDMT plus revascularization strategies might further improve the clinical outcome in HF patients with ischemic cardiomyopathy [3].

Multivessel disease (MVD) has been found in nearly half of the patients with coronary artery disease (CAD) [4–6]. Traditionally, clinical guidelines recommend coronary artery bypass grafting (CABG) as the first choice for MVD revascularization [7]. Randomized controlled trials conducted for specific scenarios, such as left main disease and low anatomic complexity, showed that percutaneous coronary intervention (PCI) is not inferior to CABG in reduction of major adverse cardiovascular events (MACEs) [8–10]. In addition, because of the advances in procedural techniques, devices, and operator experiences, PCI has become an alternative choice in the management of MVD patients with high surgical risk, such as those with HF [11].

As extensive revascularization is the main advantage of CABG over PCI, it is reasonable to pursue angiographic complete revascularization (CR) rather than culprit-only or incomplete revascularization (IR) in patients undergoing PCI instead of CABG [12]. However, angiographic CR cannot always be achieved in daily practice. A reasonable IR that is guided by anatomic, functional, and physiological parameters identifying small myocardial area at risk may be another choice [13]. However, data comparison between reasonable IR and CR in HF patients with MVD was scarce.

In this real-world prospective registry, we retrospectively analyzed outcomes in HF patients with MVD who underwent PCI either with angiographic successful CR or with residual coronary stenosis. Furthermore, we found patients with residual coronary stenosis, which may result from either initial selective IR based on clinical evidence of non-viable tissue or failed CR with clinical evidence of viable tissue. Therefore, the goal of this study was comparison of the 5-year outcomes of different PCI revascularization strategies with either successful CR, selective IR, or failed CR in HF patients with MVD.

## 2. Materials and Methods

### 2.1. Study Population, Definitions, and Clinical Follow-Up

In the present study, we enrolled HF patients with MVD who received PCI between April 2000 and February 2017 from the Cardiovascular Atherosclerosis and Percutaneous TrAnsluminal INterventions (CAPTAIN) registry [14, 15]. This study is designed retrospectively by using a single center registry database which recorded all clinical parameters prospectively. Patients with evidence of left ventricular EF <40% and stenosis >50% in at least two native coronary arteries were included. Patients who underwent bypass surgery, showed inability to tolerate dual antiplatelet therapy, and were unable to follow the study protocol were excluded. Dual antiplatelet therapy, which combined aspirin with a P2Y12 inhibitor (clopidogrel or ticagrelor), was administered to all enrolled patients for at least 9 months according to national health insurance regulations. This study complied with the Declaration of Helsinki and ethical approval for this study was obtained from the Institutional Review Board of Chang Gung Medical Foundation. All participants provided informed consent for PCI and the follow-up protocol.

**Figure 1:**
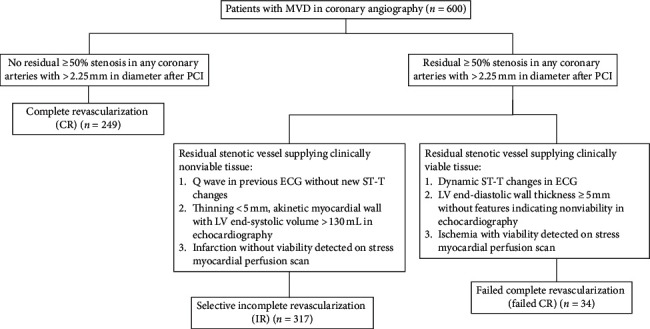
Study flow chart.

As the study flowchart in Figure 1, we finally categorized MVD patients in this study into three groups: CR, selective IR, and failed CR. Angiographic MVD was defined as the presence of ≥50% stenosis in at least two major coronary vessels or their major branches. Because the smallest stent diameter in our laboratory was 2.25 mm, the definitions of CR and IR in previous studies were modified as follows [16–18]. All patients in angiographic CR group were those without angiographic residual ≥50% stenosis in any coronary arteries with >2.25 mm in diameter regardless of viability test. Each patient in selective IR group had both viable and non-viable myocardium. Treating stenotic vessels supplying possible viable tissue and sparing stenotic vessel supplying non-viable tissue by direct or indirect viability test were performed in patients in selective IR group. Failed CR were defined as angiographic residual stenosis with viable myocardium that received failed stenotic vascular intervention. Myocardium with one of the following conditions was considered as nonviable tissue: *Q* wave in previous electrocardiogram without new ST-T changes [19]; thinning <5 mm, akinetic myocardial wall with left ventricular end-systolic volume >130 mL in echocardiography [20–22]; and infarction without viability detected on stress myocardial perfusion scan [22]. Myocardium with one of the following conditions was considered as viable tissue: dynamic ST-T changes in electrocardiogram [19]; left ventricular end-diastolic wall thickness ≥5 mm without features indicating non-viability in echocardiography [20, 22]; and ischemia with viability detected on stress myocardial perfusion scan [22]. Long-term MACEs during follow-up were defined as a composite of recurrent myocardial infarction (MI), any revascularization, and all-cause mortality in 5 years. Recurrent MI was diagnosed in cases with prolonged chest pain that lasted for more than 30 minutes, ST segment elevation or depression of at least 0.2 mV in two or more contiguous electrocardiogram leads, and significantly elevated levels of cardiac enzymes. Any revascularization was defined as further PCI or CABG after discharge from index hospitalization due to any clinical reason.

A review of all patients' medical records was conducted to obtain information on clinical status, medical management, and occurrence of any adverse event. Patients were followed up clinically in the outpatient department or through phone calls. Follow-up was scheduled at 1, 2, and 3 months after the procedure and every 3 months thereafter. The index date was defined as the date that patients underwent final PCI. Baseline characteristics were defined as the data from discharge or outpatient clinic diagnosis before the index date. Patients were followed until the first occurrence of any event, including MI, revascularization, and death after their index date for 5 years or until they completed uneventful follow-up until November 30, 2019.

### 2.2. Statistical Analysis

All results are presented as means ± standard deviation or percentages and categorical data are presented as numbers. The normality of all variables was analyzed. For continuous data, groups were compared using the *t* test or Wilcoxon rank-sum test based on the distribution. Categorical variables were compared using the chi-squared test. The clinical outcomes were compared between angiographic CR, selective IR, and failed CR groups. A multivariate Cox proportional hazards regression model was used to evaluate outcomes in patients with different revascularization strategies. Baseline characteristics and variables with *p* value < 0.2 in comparison between these three groups were adjusted in Cox proportional hazards regression analysis. To eliminate the procedure related short term effect, landmark survival analysis (index to 30 days, and 30 days to 5 years) was performed. Multivariate analysis was performed using the Cox regression model to identify independent predictors for 5-year MACEs. All results with *p* value less than 0.05 were defined as significant. Survival was investigated using the log-rank test with Kaplan-Meier curves. All statistical analyses were performed using SPSS 17.0 for Windows.

## 3. Results

### 3.1. Patient Characteristics

A total of 600 HF patients with MVD, including 249 patients with successful angiographic CR and 351 patients receiving PCI with residual coronary stenosis, were enrolled for further analysis. In patients with residual coronary stenosis, 317 patients underwent PCI with initial selective IR and 34 patients attempted CR initially but eventually failed PCI. The successful rate of angiographic CR was 88%. The reasons of failed CR included chronic total occlusion (CTO) lesion wiring failure (38.2%), inability of patient hemodynamic condition to tolerate the whole PCI procedure (35.4%), presence of an un-dilatable lesion (17.6%), and occurrence of procedure related complications (8.8%).

Baseline characteristics of the study population are presented in Table 1. In general, there was no significant difference between angiographic CR and selective IR in all clinical variables including age, sex, diabetes mellitus, hypertension, hyperlipidemia, smoking, family history of CAD, previous MI, previous stroke, acute coronary syndrome presentation, NYHA functional class, LVEF, estimated glomerular filtration rate, chronic kidney disease stage, calcified lesion, ostial lesion, bifurcation lesion, CTO lesion, use of drug-eluting stents, and use of long-term GDMT including ACEi/ARB therapy, beta-blocker therapy, and MRA. Most incidences of clinical variables in failed CR group were also similar to those in the other two groups, but patients in failed CR group had significant higher incidence of NYHA functional class 4, lower LVEF, and more CTO lesions, compared with those in angiographic CR and selective IR groups.

### 3.2. Clinical Outcomes between Successful Angiographic CR, Selective IR, and Failed CR

**Figure 2:**
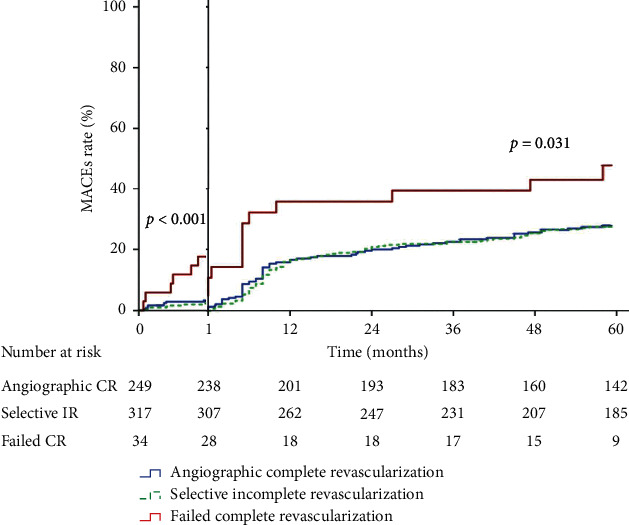
Kaplan-Meier curve of 5-year MACEs by revascularization status.

After a mean follow-up duration of 3.7 ± 1.9 years, 187 patients (31.2%) suffered from 5-year MACEs. Kaplan-Meier analysis demonstrated no difference of MACEs between angiographic CR and selective IR. A significant higher incidence of 30-day MACEs with failed CR was noted (30-day MACEs in angiographic CR, selective IR, and failed CR = 4.4%, 3.2%, and 17.6%; log-rank *p* < 0.001). Even when excluding 30-day MACEs after PCI, failed CR still had higher incidence of MACEs from 30 days to 5 years (30-day to 5-year MACEs in angiographic CR, selective IR, and failed CR = 27.8%, 27.7%, and 46.4%, respectively; log-rank *p* < 0.031) (Figure 2).

Proportional hazards regression model was performed after adjusting baseline clinical variables with *p* value < 0.2 in Table 1 including diabetes mellitus, hyperlipidemia, previous stroke, left ventricular ejection fraction, bifurcation lesion, chronic total occlusion, and NYHA functional class. The result showed failed CR was associated with higher risk of 30-day mortality (adjusted hazard ratio [HR] = 5.38; 95% confidence interval [CI]: 1.78–16.2; *p*=0.003), 5-year mortality (adjusted HR = 2.96; 95% CI: 1.51–5.81; *p*=0.002), and 5-year MACEs (adjusted HR = 2.87; 95% CI: 1.72–4.77; *p*=0.001) compared with angiographic CR (Table 2).

### 3.3. Predictors for 5-Year MACEs in HF Patients with MVD Undergoing PCI

Variables in Table 1 and revascularization strategy were adjusted for multivariate Cox regression and it revealed that hyperlipidemia (HR 1.39; 95% CI 1.03–1.86; *p*=0.031), calcified lesion (HR 1.59; 95% CI 1.17–2.17; *p*=0.003), bifurcation lesion (HR 2.52; 95% CI 1.66–3.83; *p*=0.001), ostial lesion (HR 1.63; 95% CI 1.10–2.43; *p* = 0.016), use of ACEi/ARB (HR 0.55; 95% CI 0.41–0.76; *p*=0.001), use of beta blocker (HR = 0.37; 95% CI = 0.26–0.52; *p*=0.001), and failed CR (HR = 2.59; 95% CI = 1.54–4.37; *p*=0.001, compared to angiographic CR) were independent predictors for long-term MACEs. Compared with the angiographic CR group, the selective IR group did not have significantly higher risk of long-term MACEs (HR 1.00; 95% CI 0.73–1.37; *p*=0.976) (Table 3).

## 4. Discussion

The major findings of this study are as follows: (1) in HF patients with MVD, clinical selective IR, which bypasses perfusion of clinically nonviable myocardium, had comparable long-term outcomes with angiographic CR. (2) Failed CR was significantly associated with higher risk of short-term and long-term MACEs compared to angiographic CR and selective IR in HF patients with MVD. To the best of our knowledge, this is the first study to focus on the comparison of outcomes between angiographic CR, selective IR, and failed CR in HF patients with MVD.

Most CAD patients with MVD who undergo PCI with CR rather than IR have better outcomes [23–26]. However, in certain scenarios, for example, MVD patients with cardiogenic shock, the benefit of CR is compromised because of the increased risk of acute kidney injury [27, 28]. Regarding MVD patients with HF, the Surgical Treatment for IsCHemic heart failure (STICH) study, which compared CABG to medical therapy alone, showed that the operative risk of CABG tripled the risk of overall mortality in the first 30 days and resulted in a nonsignificant difference in 2-year all-cause death between CABG and medical therapy [29]. After extending follow-up period to 10 years in STICH extension study, CABG plus optimal medical therapy had better outcomes than medical therapy alone in lower incidence of 10-year all-cause mortality. However, investigators also found presence of viable myocardium prior to surgery could not identify patients who were more likely to benefit from surgical revascularization [30, 31]. The results of STICH elucidated some controversies surrounding MVD management in HF patients, such as possible short-term procedure-related complications versus long-term hemodynamic benefits through the recovery of myocardial perfusion, using viability test guided revascularization or not. PCI was superior to CABG in periprocedural risks but inferior in CR achievement [7, 32]. But very few studies have addressed the issue of outcomes of PCI with CR versus reasonable IR guided by clinical factors in HF patients with MVD. Therefore, this study is more a comparison between the different CAD management strategies than a comparison between different angiographic results (CR or IR) in HF patients.

A recent PCI study, Chronic Heart Failure Analysis and Registry in the Tohoku District-2 (CHART-2) showed that, after a mean follow-up of 3 years, residual coronary stenosis after PCI had increased all-cause mortality in patients with mid-range or preserved EF (>40%) but not in those with reduced EF (<40%) [33], consistent with our results. However, our study provided a longer follow-up period, a greater number of HF patients with EF <40%, and data of tissue viability. Different from CHART-2 study, we also found residual coronary stenosis status could be divided into either selective IR or failed CR. Selective IR had comparable outcome with successful CR, but failed CR was associated with increased risk of MACEs. The selective IR in this study can be classified as functional guided reasonable IR [13]. Despite the fact that failed CR group was similar to selective IR group having residual coronary artery stenosis, residual stenosis vessels resulting from unsuccessful PCI procedures in failed CR group supplied moderate to large viable myocardium territory. Therefore, failed CR could not be classified as reasonable IR. Apart from disease and procedure complexity, the differences in myocardial viability of non-revascularization coronary artery may result in differences in clinical outcomes between PCI with selective IR and failed CR strategies.

There are several possible explanations why CR cannot achieve better outcomes than selective IR in HF patients. First, HF patients have abnormal coronary hemodynamics and resting myocardial energetics that may cause silent ischemia, even in the absence of CAD [34, 35]. Therefore, the benefits of CR in maintaining coronary artery patency angiographically may be limited by the existence of microvascular or silent ischemia [36]. Second, according to previous studies, iatrogenic infarction occurs in nearly 30% of revascularization procedures in both percutaneous and surgical interventions [37]. In addition, either the prolonged procedure times or more complex interventions required in CR increase the risk of periprocedural infarction and may offset the benefit from improved myocardial perfusion. Third, short-term risks, including contrast-induced nephropathy associated with a higher dose of contrast medium and stent thrombosis from multiple stenting, may outweigh the potential long-term benefits associated with CR.

Although PCI with selective IR in this study seemed to be a conservative strategy for managing MVD in HF patients, the principle of clinical selective IR is revascularization with the goal to avoid revascularization in nonviable myocardium that presented as infarction on perfusion scanning; wall thinning, fibrosis, or scar formation on echocardiography; or pathologic *Q* wave without ST segment change on electrocardiography. Angiographic CR in our study, in contrast, is a more aggressive strategy to achieve complete angiography-guided revascularization. In certain clinical scenarios, an aggressive strategy does not necessarily mean a good outcome because patients have to take a risk. As shown in our study, the incidence of failed CR was only 12% in all patients attempting angiographic CR, but the risk of MACEs was 2.6-fold higher in the failed CR group than in the successful CR and selective IR groups. Conclusively, more comprehensive evaluation and planning are required before PCI for HF patients with MVD. If CR is technically difficult, selective, or viable, tissue-guided IR may be an alternative option.

There are several limitations in this study. First, the definition of CR was based on anatomic criteria instead of functional criteria (treatment of all coronary segments >1.5 mm with fractional flow reserve <0.80). Second, the viability evaluation was based on clinical evidence and stress myocardial perfusion scan whereas cardiac magnetic resonance imaging which is a gold standard for assessment of myocardial viability is not used. Third, this study had an observational design and was based on a real-world registry. Although the confounding factors were considered and adjusted for, the confounding effects may have biased our results. Third, the selection of the target vessel in the IR group was mainly based on the physician's judgment according to coronary angiography, electrocardiography, echocardiography, and myocardial perfusion scan but not according to magnetic resonance imaging or fractional flow reserve study. To further quantify the viable myocardium in HF patients with MVD, additional large, prospective, randomized studies are required in the future.

## 5. Conclusion

During a mean follow-up period of 3.7 ± 1.9 years, HF patients undergoing PCI with selective IR had no significant difference in long-term MACEs compared to successful CR. However, failed CR had higher risk of MACEs than selective IR and successful CR. These results indicate that a more comprehensive planning strategy should be devised before PCI in HF patients with MVD. If the risk of failed CR outweighs the benefit of CR in HF patients with MVD, selective IR may be an alternative option.

## Figures and Tables

**Table 1:** Baseline characteristics of heart failure patients with multivessel disease according to final angiographic results.

	Angiographic CR (1)	Selective IR (2)	Failed CR (3)	*p* value for (1) vs. (2)	*p* value for (1) vs. (3)	*p* value for (2) vs. (3)
Patient number, n	249	317	34			
Age, years	64.4 ± 11.7	64.4 ± 11.9	63.8 ± 12.8	0.963	0.762	0.778
Male sex, *n* (%)	209 (83.9)	255 (80.4)	28 (82.4)	0.322	0.806	1.000
Diabetes mellitus, *n* (%)	107 (43.0)	156 (49.2)	14 (41.2)	0.149	1.000	0.471
Hypertension, *n* (%)	135 (54.2)	187 (59.0)	22 (64.7)	0.267	0.274	0.584
Hyperlipidemia, *n* (%)	104 (41.8)	149 (47.0)	19 (55.9)	0.233	0.141	0.369
Smoking, *n* (%)	108 (43.4)	129 (40.7)	16 (47.1)	0.548	0.715	0.471
Family history of CAD, *n* (%)	4 (1.6)	4 (1.3)	0 (0.0)	0.736	1.000	1.000
Previous history of MI, *n* (%)	193 (77.5)	254 (80.1)	25 (73.5)	0.468	0.664	0.374
Previous stroke, *n* (%)	8 (3.2)	21 (6.6)	3 (8.8)	0.084	0.133	0.717
NYHA Fc						
Class I, *n* (%)	112 (45.0)	127 (40.1)	8 (23.5)	0.265	0.025	0.065
Class II, *n* (%)	68 (27.3)	83 (26.2)	8 (23.5)	0.775	0.837	0.839
Class III, *n* (%)	41 (16.5)	67 (21.1)	8 (23.5)	0.196	0.334	0.826
Class IV, *n* (%)	28 (11.2)	40 (12.6)	10 (29.5)	0.696	0.007	0.017
ACS, *n* (%)	101 (40.6)	116 (36.6)	15 (44.1)	0.340	0.713	0.456
eGFR, ml/min/1.73m2	71.5 ± 29.6	67.4 ± 26.2	65.0 ± 26.7	0.358	0.256	0.838
CKD stage >3, *n* (%)	89 (35.7)	132 (41.6)	15 (44.1)	0.165	0.349	0.855
LVEF, %	32.1 ± 6.8	32.2 ± 7.0	29.1 ± 8.2	0.821	0.015	0.020
Calcified lesion, *n* (%)	67 (26.9)	81 (25.6)	7 (20.6)	0.773	0.535	0.678
Ostial lesion, *n* (%)	34 (13.7)	43 (13.6)	3 (8.8)	1.000	0.591	0.596
Bifurcation, *n* (%)	26 (10.4)	25(7.9)	0(0.0)	0.304	0.054	0.152
Chronic total occlusion, *n* (%)	36 (14.5)	35 (11.0)	14 (41.2)	0.250	<0.001	<0.001
Drug-eluting stenting, *n* (%)	138 (55.4)	166 (52.4)	16 (47.1)	0.497	0.366	0.592
Survival to discharge, m	238	307	26			
Use of ACEi/ARB, *n* (n/m%)	191 (80.3)	248 (80.8)	20 (76.9)	0.913	0.617	0.610
Use of beta blocker, *n* (n/m%)	215 (90.3)	279 (90.9)	22 (84.6)	0.883	0.319	0.296
Use of MRA, *n* (n/m%)	46 (19.3)	62 (20.2)	3 (11.5)	0.829	0.433	0.439

ACEi, angiotensin-converting enzyme inhibitors; ACS, acute coronary syndrome; ARB, angiotensin receptor blockers; CAD, coronary artery disease; CKD, chronic kidney disease; CR, complete revascularization; eGFR, estimated glomerular filtration rate; IR, incomplete revascularization; LVEF, left ventricular ejection fraction; MI, myocardial infarction; MRA, mineralocorticoid receptor antagonist; NYHA Fc, New York Heart Association Functional classification; RCS, residual coronary stenosis.

**Table 2:** Five-year follow-up outcomes in patients with heart failure with multivessel disease according to revascularization strategies.

Variables	Patient number, *n*	Events, *n* (%)	Crude HR (95% CI)	*p* value	Adjusted^#^ HR (95% CI)	*p* value
*Recurrent MI*						
Angiographic CR	249	20 (8.0)	1.00 (reference)	—	1.00 (reference)	—
Selective IR	317	16 (5.0)	0.62 (0.32–1.20)	0.157	0.65 (0.34–1.26)	0.653
Failed CR	34	1 (2.9)	0.50 (0.07–3.72)	0.498	0.57 (0.08–4.24)	0.579
*Any revascularization*						
Angiographic CR	249	38 (15.3)	1.00 (reference)	—	1.00 (reference)	—
Selective IR	317	54 (17.0)	1.10 (0.73–1.66)	0.660	1.13 (0.75–1.72)	0.556
Failed CR	34	7 (20.6)	1.94 (0.87–4.35)	0.106	2.12 (0.94–4.75)	0.069
*30-day mortality*						
Angiographic CR	249	8 (3.2)	1.00 (reference)	—	1.00 (reference)	—
Selective IR	317	7 (2.2)	0.68 (0.25–1.88)	0.457	0.59 (0.21–1.63)	0.304
Failed CR	34	6 (17.6)	5.80 (2.01–16.7)	0.001^*∗*^	5.38 (1.78–16.2)	0.003^*∗*^
*5-year mortality*						
Angiographic CR	249	34 (13.7)	1.00 (reference)	—	1.00 (reference)	—
Selective IR	317	41 (12.9)	0.94 (0.60–1.48)	0.784	0.90 (0.57–1.42)	0.641
Failed CR	34	12 (35.3)	3.34 (1.73–6.45)	0.001^*∗*^	2.96 (1.51–5.81)	0.002^*∗*^
*5-year MACEs*						
Angiographic CR	249	75 (30.1)	1.00 (reference)	—	1.00 (reference)	—
Selective IR	317	93 (29.3)	0.96 (0.71–1.30)	0.801	1.01 (0.74–1.37)	0.962
Failed CR	34	19 (55.9)	2.53 (1.53–4.19)	0.001^*∗*^	2.87 (1.72–4.77)	0.001^*∗*^

^#^Adjust baseline clinical variables with *p* value < 0.2 in Table 1 including diabetes mellitus, hyperlipidemia, previous stroke, left ventricular ejection fraction, bifurcation lesion, chronic total occlusion, and NYHA Functional Class in the Cox proportional regression model. ^*∗*^*p* value <0.05. Definition of MACEs: composite of myocardial infarction, revascularization, in-hospital mortality, and all-cause mortality. CR = complete revascularization; IR = incomplete revascularization; MACEs = major adverse cardiac events; MI = myocardial infarction.

**Table 3:** Predictors of 5-year MACEs in HF patients with MVD undergoing PCI.

Variables	HR (95% CI)	*p* value
Hyperlipidemia	1.39 (1.03–1.86)	0.031
Calcified lesion	1.59 (1.17–2.17)	0.003
Bifurcation lesion	2.52 (1.66–3.83)	0.001
Ostial lesion	1.63 (1.10–2.43)	0.016
Use of ACEi/ARB	0.55 (0.41–0.76)	0.001
Use of beta blocker	0.37 (0.26–0.52)	0.001

Revascularization strategy		
CR (reference)	1.00 (reference)	—
Selective IR	1.00 (0.73–1.37)	0.976
Failed CR	2.59 (1.54–4.37)	0.001

ACEi, angiotensin-converting enzyme inhibitors; ARB, angiotensin receptor blockers; CI, confidence interval; CR, complete revascularization; HR, hazard ratio; IR, incomplete revascularization.

## Data Availability

The data used to support the findings of this study are restricted by the regulation of Institutional Review Board in order to protect patient privacy.
